# Exploring Environmental Triggers and Viral Associations in a Case of Severe Bullous Pemphigoid

**DOI:** 10.7759/cureus.83266

**Published:** 2025-04-30

**Authors:** Alexandra Lawlor, Rachel Alef, Khaled Deeb

**Affiliations:** 1 Rheumatology, Kansas City University College of Osteopathic Medicine, Kansas City, USA; 2 Osteopathic Medicine, Nova Southeastern University Dr. Kiran C. Patel College of Osteopathic Medicine, Davie, USA; 3 Internal Medicine, The Veteran Affairs West Palm Beach (VA WPB), West Palm Beach, USA

**Keywords:** autoimmune disease, bullous pemphigoid, dermatology, environmental exposures, rheumatology, viral associations

## Abstract

Bullous pemphigoid (BP) is an autoimmune disease characterized by pruritic blisters and plaques, primarily affecting elderly individuals. The etiology of BP is multifactorial, with several documented triggers, the extent of which is currently unknown. This case reviews currently documented BP triggers and presents a case of severe and refractory BP, whose onset and progression raise suspicion for indoor pollutants and viral infection as inciting triggers. Our patient's condition worsened despite standard therapy, highlighting the complexity of BP management. This case adds to the literature, teasing out BP pathogenesis and highlighting the complicated hospital treatment course for severe autoimmune blistering diseases.

## Introduction

Bullous pemphigoid (BP) is an uncommon autoimmune disease characterized by pruritic blisters and urticarial plaques, primarily affecting elderly individuals [1]. The disease manifests through a complex interplay of immunological factors and tissue destruction mechanisms. Pathologically, BP is characterized by eosinophilic infiltration with subepidermal blister formation, attributed to autoantibodies targeting hemidesmosomal proteins within the basement membrane zone [2]. This autoimmune response triggers complement activation and recruitment of inflammatory cells, contributing to blister formation and skin inflammation [3].

In order to diagnose this complex autoimmune disease, eosinophils with subepidermal detachment should be seen on histopathology, C3 and/or IgG deposition at basement membranes should be seen on immunofluorescence assays, and circulating autoantibodies against hemidesmosomal anchoring proteins should be measured via enzyme-linked immunosorbent assay (ELISA) [1]. Treatment of bullous pemphigoid classically involves topical and systemic steroids, and certain studies have demonstrated the usefulness of an adjunctive therapy using doxycycline, dapsone, and immunosuppressants [1]. 

While the precise trigger for BP remains elusive, various factors are implicated in its pathogenesis. Genetic predisposition, coupled with environmental triggers, may initiate tissue damage and subsequent autoimmune response [4]. These triggers encompass a spectrum of agents, including drugs, vaccines, viral, bacterial, and parasitic infections (herpes viruses, human immunodeficiency virus (HIV), Helicobacter pylori, group A streptococcus infections, Sarcoptes scabiei, etc.), ultraviolet phototherapy, and physical phenomena such as surgery or trauma [4,5]. 

The intricate interplay between genetic susceptibility, environmental factors, and immune dysregulation underscores the complexity of BP etiology. This case report aims to explore the potential links between indoor pollutants, respiratory syncytial virus (RSV) infection, autoantibodies, and BP exacerbation, shedding light on novel aspects of the disease etiology and management.

## Case presentation

A 67-year-old male with a history of asthma presented to the emergency department in December 2023 with a worsening bullous rash that was unresponsive to steroid therapy. In early September, the patient first noticed small, non-bullous, pruritic lesions on his extremities. These lesions gradually increased in size, became bullous, and spread to his trunk, groin, scalp, and oral mucosa.

Despite treatment with topical agents, the condition continued to progress. By mid-November, he was prescribed clindamycin, famotidine, loratadine, and methylprednisolone (4 mg four times per day (QID) for six days), but the rash persisted. In late November, the patient developed flu-like symptoms and tested positive for respiratory syncytial virus (RSV), which corresponded with a noticeable exacerbation of his skin condition. The bullae enlarged, the rash spread, and pruritus increased.

**Figure 1:**
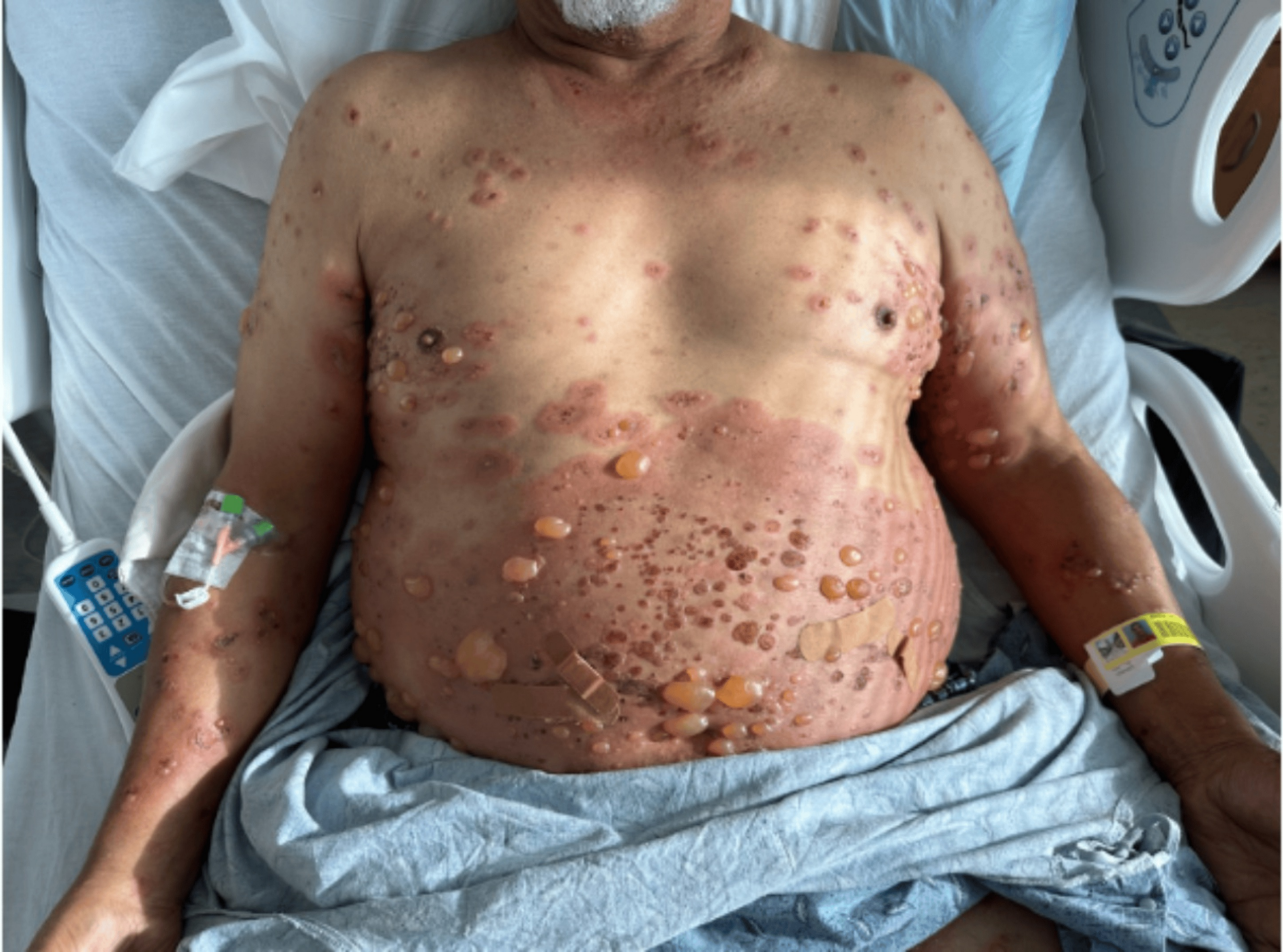
Bullous pemphigoid on the patient's trunk The patient's trunk and upper extremities demonstrate confluent, moderate-to-severe erythematous plaques with overlying tense, straw-colored 0.2 to 2.0 cm bullous lesions. Blisters and pruritic papules can be noted throughout the rash. Larger bullous lesions were tender to palpation, and smaller papular to vesicular lesions were severely pruritic (worst on the plantar-palmar surfaces, not shown in this figure). Nikolsky's sign was negative.

**Figure 2:**
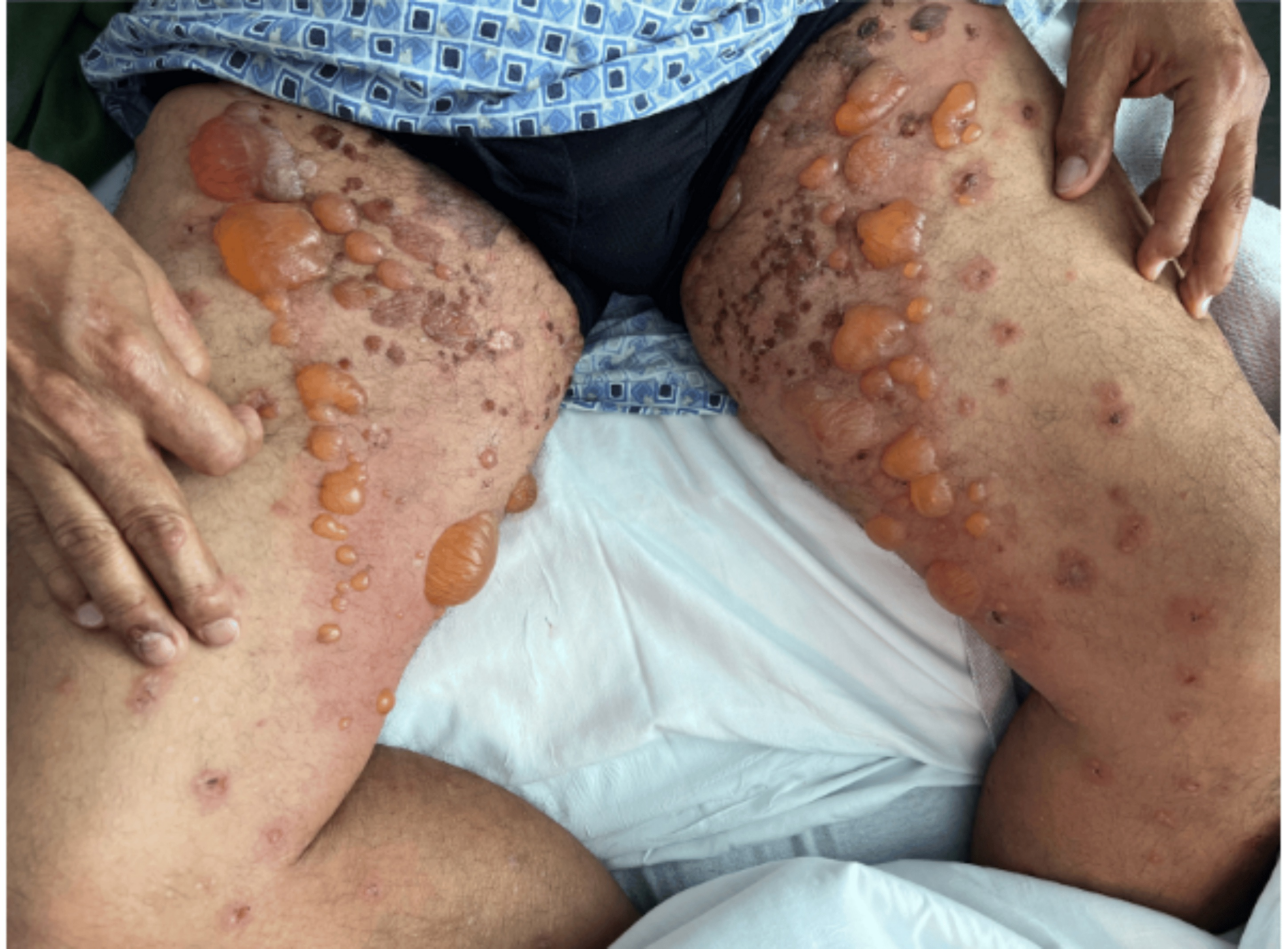
Bullous pemphigoid on patient's proximal extremeties The patient's proximal lower extremities demonstrate the same confluent, moderate-to-severe erythematous plaques with overlying tense, straw-colored 0.2 to 2.0 cm bullous lesions as shown on trunk and upper extremities. Blisters and pruritic papules can again be noted throughout the rash. Nikolsky's sign was negative.

Upon admission to the hospital in early December 2023, he exhibited confluent erythematous plaques with overlying tense bullae ranging from 0.2 to 2.0 cm. These plaques extended across his trunk (severity can be noted in Figure 1) and proximal extremities (severity can be noted in Figure 2), with blisters and pruritic papules scattered throughout the rash. He also noticed involvement of his palms, soles, forehead, scalp, and oral mucosa. Pruritus was severe enough to interfere with sleep. Despite these significant symptoms, he was hemodynamically stable and did not report fever, chills, nausea, or vomiting, although he had experienced a weight loss of 12 to 15 pounds over the previous two to three months.

The patient was admitted for a total of five days. Throughout the admission, the patient was hemodynamically stable and continued to deny constitutional symptoms. Laboratory tests on admission showed initial neutrophilia and eventual eosinophilia, indicating a complex immune response. Considering the severe presentation and range of differential diagnoses, including bullous pemphigoid, pemphigus vulgaris, linear IgA bullous dermatosis, dermatitis herpetiformis, and herpes simplex dermatitis, clinicians opted for an aggressive treatment approach. In the hospital, he was treated with intravenous immunoglobulin (IVIG) 40 gm 400 mL for the entirety of hospitalization (one bottle per day); high-dose prednisone (60 mg by mouth (PO) day 0 85 mg PO days 1 to 4, and 60 mg PO on day 5; doxycycline 100 mg twice per day PO starting day 1 to 5); valacyclovir 1000 mg every eight hours PO starting day 2 to 5; hydroxyzine pamoate 20 mg PO on day 0, 50 mg PO three times daily on days 1 to 3 (discontinued mid-day on day 3), 50 mg PO every four hours for part of (midday) day 3, 50 mg PO every six hours on day 3 evening through day 5); loratadine 10 mg PO each day for the entirety of hospitalization; montelukast 10 mg PO each night for the entirety of hospitalization; and various topical creams (small amount of topical calamine lotion twice per day for the entirety of hospitalization, small amount of topical clobetasol proprionate 0.5% ointment 30 gm days 0 to 3 of hospitalization, small amount of topical hydrocortisone 1% cream 30 gm twice per day on days 3 to 5, topical triamcinolone acetonide 0.025% cream 15 gm given twice per day given on days 3 to 5). Note: the treatment with dapsone was also initiated but discontinued following identification of glucose-6-phosphate dehydrogenase deficiency.

The patient received testing and evaluation for malignancy, celiac disease, hepatitis, syphilis, herpes simplex virus (HSV), HIV, and parasites, all of which were negative. The patient was ultimately diagnosed with BP due to immunofluorescence microscopy of three biopsy specimens, revealing linear deposition of immunoglobulin G and complement component C3 along the basement membrane zone, confirming a diagnosis of BP. The patient additionally had positive BP180 (results = 168 RU/mL, normal value < 15 RU/mL) and BP230 (results = 64 RU/mL, normal value < 9 RU/mL) antibodies. Over the course of hospitalization, the patient’s condition remained stable, with some mild improvement in pruritus, and the patient reported being able to sleep multiple hours a night for the majority of the nights. Additionally, there was mild improvement in rash, most notable for decreased erythema of the underlying plaques. The patient was discharged on prednisone 85 mg taper, doxycycline 100 mg PO twice per day, valacyclovir 100 mg PO every eight hours, hydroxyzine pamoate 50 mg PO every six hours, loratidine 10 mg PO, montelukast 10 mg PO, and topical creams (same as above) with a follow-up dermatology appointment post-discharge. 

At a follow-up dermatology appointment one day after discharge, the patient’s rash worsened, and he reported being unable to sleep due to severe pruritus, so he was started on cyclosporine (600 mg PO daily). At a follow-up dermatology appointment four days after discharge, the patient’s condition continued to worsen (1 to 2.5 cm bullous lesions) and new lesions were noted on the superior chest, neck, and penis. He was started on rituximab (1 gm IV infusions) due to cyclosporine's lack of response. 

## Discussion

Due to a lack of response during hospitalization, the team sought to identify the underlying trigger and found the patient's onset of symptoms and progression aligned with a possible environmental connection. The patient purchased a home constructed in the 1970s and undertook repairs involving drywall work and painting, suggesting possible exposure to indoor pollutants like volatile organic compounds and particulate matter, both of which have been linked to autoimmune responses [2,6]. The patient noted the onset of symptoms approximately one week thereafter. The patient’s symptoms continued to progress upon residing in the home until acute exacerbation following an RSV infection, including rapid spread of bullae and an increase in pruritus. This exacerbation highlights the potential interaction between viral infections and autoimmune processes.

While literature on RSV's impact on BP is limited, there is growing evidence that other viral infections can trigger or amplify autoimmune responses [4]. Respiratory syncytial virus elicits robust inflammatory responses characterized by the release of pro-inflammatory cytokines, such as interleukin-6 (IL-6) and tumor necrosis factor-alpha (TNF-α), which could exacerbate autoimmune-mediated tissue damage in BP. While it is possible this patient's BP could have been idiopathic, the possible influence of negative environmental exposure and RSV infection is interesting, and based upon the current model of autoimmune diseases, it is feasible these factors may have contributed to this patient's condition. Bullous pemphigoid is a multifactorial disease, and therefore, it is difficult to pinpoint exact pathogenesis factors. Possible triggers, such as home contaminants and RSV, should be further studied to determine a clearer association. 

The current model of autoimmune diseases posits that genetically susceptible individuals develop inflammatory responses to environmental triggers, which then induce B and/or T cell autoreactivity through mechanisms such as the hidden antigen theory or molecular mimicry [7]. These theories have been implicated in the pathogenesis of many autoimmune diseases, including dermatologic diseases such as dermatomyositis and psoriasis [7,8]. However, the number of kinds of triggering factors remains unknown. Previously documented environmental insults include infections, drugs, vaccinations, and physical phenomena such as surgery, trauma, UV burns, and even living near a wastewater treatment plant [2,6]. Our findings suggest that indoor pollutants should also be explored as triggering factors for BP.

Finally, consistent with the Healthy People 2030 initiative, one's neighborhood and built environment remain an important social determinant of health [9]. Thus, multidisciplinary approaches between dermatologists and public health professionals to further elucidate the relationship between environmental factors and autoimmune disease processes represents an opportunity to increase quality of life and decrease health disparities.

## Conclusions

Clinicians should consider a comprehensive approach that includes a thorough assessment of the patient's living environment and a detailed history of recent infections for patients with new-onset BP to identify an underlying trigger. If an environmental trigger is identified, it is possible that removal of that trigger could result in a reduction of symptoms due to removal of the nidus. Avenues for future research into the role of indoor pollutants and viral infections in the onset and exacerbation of autoimmune diseases like BP should be explored. By understanding these connections, clinicians can improve patient outcomes through tailored interventions and proactive management strategies, ensuring comprehensive care for individuals with refractory BP.
